# Supplementary figures and images for: Navigation task and action space drive the emergence of egocentric and allocentric spatial representations
Source: PLoS Comput Biol. 2022 Oct 31;18(10):e1010320. doi: 10.1371/journal.pcbi.1010320 (PMC9648855; doi:10.1371/journal.pcbi.1010320)

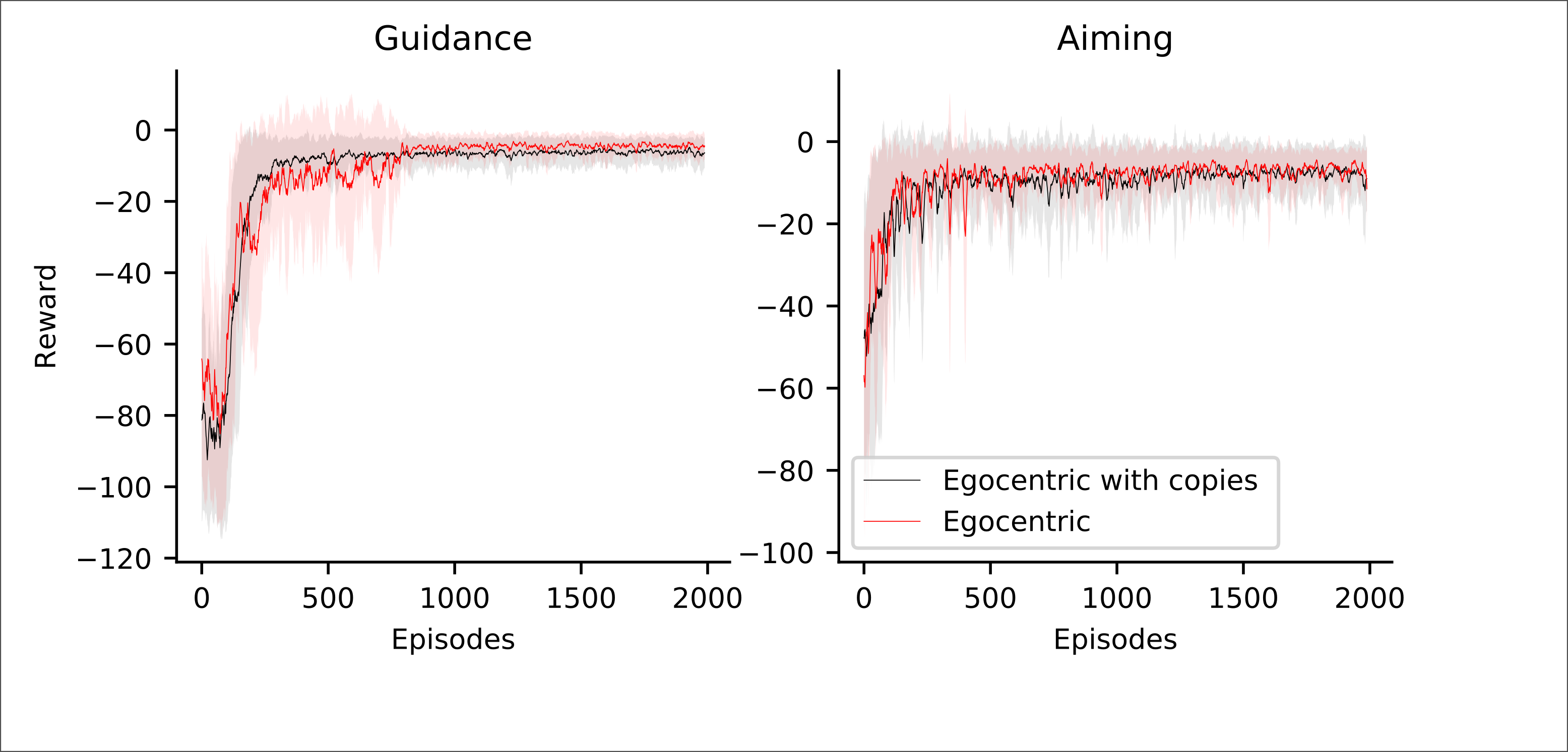

Supplement: S1 Fig — (TIFF) [file pcbi.1010320.s001.tiff]

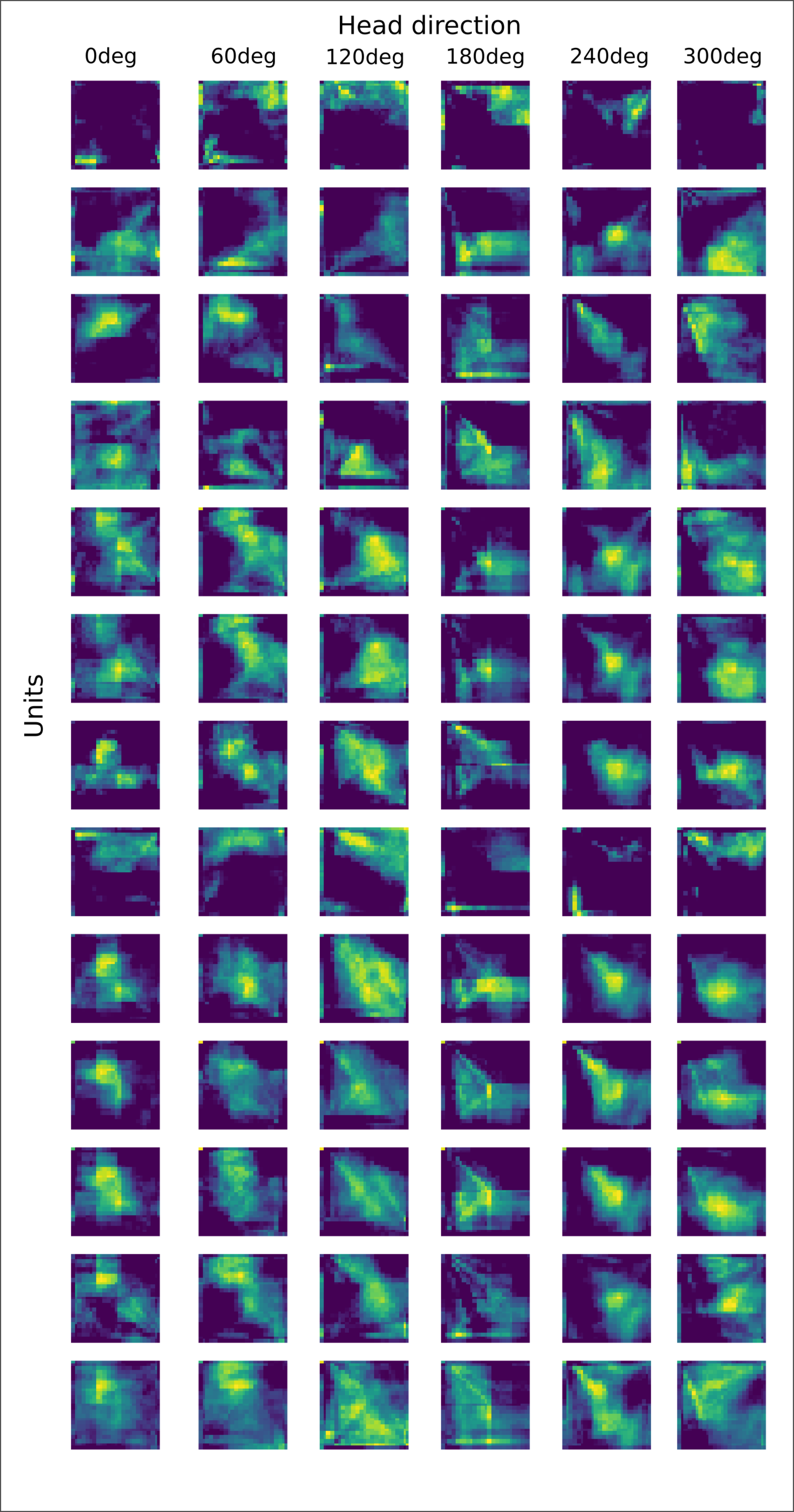

Supplement: S2 Fig — (TIFF) [file pcbi.1010320.s002.tiff]
