# Supplementary material for: Navigation task and action space drive the emergence of egocentric and allocentric spatial representations
Source: PLoS Comput Biol. 2022 Oct 31;18(10):e1010320. doi: 10.1371/journal.pcbi.1010320 (PMC9648855; doi:10.1371/journal.pcbi.1010320)
Supplement: Attachment — Submitted filename: Responses.pdf [file pcbi.1010320.s003.pdf]

We thank the reviewers for their comments and appreciate the time and efforts they spend on providing feedback and helping us improve this manuscript. Based on the suggestions and clarifications requested by the reviewers, we have made several changes to the manuscript, including clearer descriptions of aspects of the methods and results, and a more extensive discussion of relevant literature. Below are our point-by-point responses to the reviewers' comments.

Reviewer #1:

This study presents a model of learning allo- and egocentric navigation strategies using deep reinforcement learning (RL). The model takes the shape of a DQN that was allowed to take actions defined in egocentric space or actions defined in an allocentric space. The authors trained the model on two different navigational tasks, guidance and aiming, which are generally associated with allo- and egocentric navigation, respectively. Consistent with these intuitions, the model preferentially chooses allocentric actions during guidance and egocentric actions during aiming. In the final, fully connected layer of the network, place cell-like representations were found primarily during allocentric navigation, while egocentric vector representations were found more during egocentric navigation.

This study provides a valuable contribution to the literature on allocentric and egocentric navigation strategies. By using a deep network trained directly on visual inputs, the authors are able to show how both egocentric and allocentric strategies can be used to solve tasks that are associated with one or the other strategy, which is similar to experimental observations. Furthermore, their model allows ablation experiments showing the specific contributions of different cell types and highlights that differences in strategies might only be detected when looking at generalisation performance. There are thus many strengths to this manuscript. However, I think there are some important points to be addressed, see below.

1. I wonder how fairly the action spaces defined for the two models really reflect egocentric and allocentric "strategies", rather than a difference in sensory and motor affordances. The allocentric actions comprise all six movement directions as well as turning around to any of the six directions, whereas the egocentric actions are only forward, left and right. The authors get around the imbalance in the number of actions by copying the egocentric actions four times but I think there are a couple of issues that remain with this modelling approach.

(a) A defining characteristic of allocentric navigation strategies is that allocentric directions still need to be transformed into the egocentric reference frame before movement. The authors mention in the discussion that we should interpret the actions in the model more as "higher-level navigation decisions" which still have to be translated into motor commands. I'm not so convinced by this argument, because if that is the case, shouldn't that be the same for the egocentric actions too? Or are those lower-level decisions?

We fully share the reviewer's initution and also consider the egocentric actions in our model to be higher-level navigation decisions, which would have to be converted to motor commands, similar to those from allocentric actions. While at first glance it might seem to be trivial to convert egocentric navigation decisions such as "turn left" to motor commands, this might not really be the case since motor commands are ultimately executed by means of muscle activations. This transformation to muscle activations is not straightforward, depending on posture and other factors (Andersen, 1993). In particular, depending on the mode of navigation, the same higher-level navigation decisions might need to be transformed to vastly different motor commands depending on the situation, since "turn left" while walking is very different

from when driving a car or riding a bicycle. Thus, even though intuitions might suggest otherwise egocentric navigation commands are not different from allocentric ones in that they are high-level decisions, which need to be transformed into lower-level movement commands.

We added to our discussion to make this point more explicit:

(p.12 line 430ff.) *....However, even the egocentric actions outputted by our model have to be translated by a downstream system into motor commands. This is because ultimately all navigation decisions have to be transformed into the coordinates of the relevant muscles for movement. This means that the same higher-level navigation decisions may require very different coordinate transformations based on the current posture and method of navigation (e.g. "turn left" on a bicycle vs. while walking).*

Why can the egocentric agent not perform the action of turning around or walking backwards? These differences are not really the essence of egocentric and allocentric navigation but they will result in very different types of movement trajectories, where the egocentric agent needs to make a bigger circle.

There may have been a misunderstanding. In fact, both the egocentric and allocentric agents turn in place. The difference is that the allocentric action space turns the agent to face a specific direction relative to an allocentric coordinate frame *regardless* of its original heading direction, while the egocentric turning actions turn the agent left or right *relative* to its current heading direction. Thus, the egocentric agent can effectively turn around in place by executing a sequence of left or right turns. To clarify this aspect of the egocentric agent, we have expanded the text in the Methods to include a more detailed description of its action space :

(p.5 line 157)*...in the egocentric action space, the agent could choose to move forward from its current position by a fixed distance or turn in place by a fixed angle of 60 degrees either to the left or right of the current heading direction, similarly corresponding to navigating on the same hexagonal grid. The egocentric agent must thus successively make three left, or right, turns to change its orientation by 180 degrees and turn around completely.*

For the reasons mentioned above, while it is true that the egocentric agent might need to take a few extra actions to get to the goal, this difference is small, and does not lead to much longer trajectories, as seen in the reward curves. This also is evidenced by the fact that the egocentric action space is not at a significant disadvantage compared to the allocentric space, since it is chosen preferentially when it is appropriate for the task, i.e. in aiming.

We believe that this also addresses point 1(c) below, since the egocentric agent is indeed capable of turning in place.

(b) The allocentric agent can turn around on the spot and even walk backwards towards a goal. As far as I can see that strategy would be as good as turning around and walking forward to a goal, right?

It is true that sections of the trajectories of the allocentric agent could involve the agent moving in a direction it is not currently facing, including backwards, and this is a limitation of our approach. However, we chose not to restrict the allocentric agent to only be able to move forward as this imposes an egocentric bias on the agent, which we wish to avoid. In addition, “walking backward” is not explicitly provided as an action to either the egocentric or allocentric agent, because natural goal-directed navigation in mammals generally does not involve this behavior.

(c) Have the authors tried making all movement directions, including the turning actions, available to the egocentric agent? Or alternatively, restricting the allocentric agent's movement directions?

We clarified this point above in response to 1(a) — the egocentric turning actions turn the agent in place, such that the egocentric agent does not necessarily need to take much longer trajectories than the allocentric agent to reach the goal.

(d) It was unclear to me whether, when an egocentric action was chosen in the full model and a next state, reward pair was observed, all four action copies or only the chosen copy was assigned credit to. In the latter case, wouldn't a different bias arise between the two strategies as a result of slower, noisier learning in the egocentric model? In the former case, would a bias still arise because backpropagated errors for egocentric actions have 4x the effect on weight changes in the network compared to allocentric actions?

This is an important point. In our model, only the chosen action copy was assigned credit to. The reviewer is right that this might lead to noisier learning for the egocentric actions. However, in practice this difference is not large enough to put the egocentric action space at a disadvantage compared to the allocentric agent, since it is still clearly chosen when appropriate, such as in the aiming task.

However, to fully address this point, we simulated the egocentric agent with repeated actions and compared it to one without repeated actions on both guidance and aiming. The simulations showed no discernible negative effects on learning when using multiple action copies (Fig. S1). In particular, the learning curves do not show larger variability, which is what one would expect if learning were more unstable. We added :

(p.5 line 167ff.) *During training, credit was assigned only to the particular copy of the action that was chosen, not to all four copies of that action. We assessed whether this design choice made learning in the egocentric agent noisier by comparing learning curves in both tasks using either single or multiple copies of each action and found no discernible effects (Fig. S1).*

(2) The authors rightly point out that there is a considerable overlap in regions involved in egocentric and allocentric navigation strategies. There is indeed evidence for both the striatum and the hippocampus being involved in egocentric and allocentric strategies. However, the authors fail to discuss several seminal papers that do show a clear dissociation between these regions.
(a) For example, Packard & McGaugh (1996) showed that hippocampal lesions affect (allocentric) place learning while lesions to the caudate affect learning of (egocentric) responses to cues. Pearce et al (1998) showed that hippocampal lesions selectively affect place learning in the water maze. It would be great if the authors could include a more extensive discussion of this literature.

We thank the reviewer for drawing our attention to this point and literature, and believe this adds a valuable discussion to our study. Many studies on the neural basis of navigational strategies focus on the dissociation between place learning and response learning. "Place learning" aligns with what we refer to as "allocentric strategy". "Response learning" refers to the learning of habitual responses in spatial navigation and since habitual responses are typically formed in an egocentric reference frame, there is an overlap between the two. However, we believe that it is important to clearly distinguish between the use

of an egocentric navigation strategy and habitual responses. Non-habitual egocentric navigation has received less attention. Higher-level navigation decisions and planning can be done in an egocentric frame of reference as well, and we believe our study also points out the importance of studying the difference between reference frames at this level, for example, by studying generalization. We added a more extensive discussion of this point including the suggested literature in our revised manuscript (see below, after 2(c)).

(b) Do the authors think there is no modularity at all in egocentric and allocentric systems, or is there a softer form of modularity than previously assumed?

As we hypothesize in the Discussions, at more abstract, cognitive levels that are responsible for decision making and planning in navigation, we believe that there is likely less modularity in allocentric and egocentric systems than previously assumed. However, prompted by the reviewer we reflected further on this question and concluded that modularity might be different for different computational levels and functions. This might help resolve the conflicting experimental evidence wherein some studies show a clear dissociation between areas using allocentric and egocentric reference frames and others suggesting overlapping regions . For instance, systems responsible for habitual response learning in the egocentric reference frame or for coordinate transformations are more likely to be modular. We added a more extensive discussion of this point in our revised manuscript (see below, after 2(c)).

(c) What are the computational benefits and disadvantages of a modular architecture, and how does this relate to the present model?

Modular systems are more specialized and therefore faster and computationally less intensive, which lends itself well to simpler and repetitive tasks. At higher, more abstract levels, however, this becomes less useful, and flexibility, robustness and generalization are arguably more important. This type of system would need to be able to use both allocentric and egocentric frames of reference depending on the task at hand, and even flexibly switch between the two if needed. We conceive of our model at this higher level, although we think it is an interesting question for future work to study the use of reference frames at these two different levels and model their interactions.

To address points 2(a-c), we added the following text to our discussion:

(p.14 line 519) *In a similar vein, our work also adds some perspective to the place vs. response literature (Goodman, 2021).*

Response based navigation is inherently egocentric in nature and typically develops with repeated experiences of the same route (Hicks, 1964; Hicks, 1999; Hicks, 1996; Hicks, 1950). The dorsal striatum and specifically the caudate nucleus have been implicated in the development of these habitual responses (Packard, 1996). While the tasks used in these dissociation studies indeed evoke the use of an allocentric or egocentric reference frame and can pinpoint the corresponding neural basis, there is an added layer of complexity because these reference frames are also being used at distinct levels - a more abstract, cognitive level associated with the place strategy, and the rote, habitual level associated with the response strategy.

We believe that our study brings attention to the relatively understudied use of the egocentric reference frame in higher-level navigation planning and decisions, which might still depend on the hippocampus, thus leading to conflicting experimental evidence. This might explain why, for relatively simpler tasks,

hippocampal animals are still able to use egocentric navigation strategies, but for more complex tasks, lesions to the hippocampus seem to affect both strategies (Fouquet, 2013).

Experimental results are also consistent with the suggestion that while regions at the lower, specialised levels of the navigation system are likely modular, such as the coordinate transformation system in the retrosplenial cortex (Clark, 2018) and habitual responses in the dorsal striatum (Packard, 1996), there may be less modularity in the higher, more general levels than previously assumed. We liken our model to an abstract higher-level navigation system which would need to be flexible and lend itself well to generalization, including being able to use and represent both frames of reference depending on the task.

(3) The authors' hypotheses and simulations about generalisation were to me some of the most interesting in the paper. However I missed a few things in that section, which might be just an issue with exposition.
(a) It is unclear to me what exactly is shown in figures 7B&D. Is it the proportion of successful trials after having been trained from different starting points (B) or different goal locations (D)? This seems implied for 7B but is not very clear for 7D. Please clarify.

Both Figs. 7B and 7D show the performance on the test trials after the agent has been fully trained. The training and testing varied between the two tasks due to their different inherent structure. In the guidance task, i.e. Fig. 7B, the agent was trained to navigate to an unmarked goal location from different starting points and during test had to navigate to the same goal location from novel starting points. In the aiming task, i.e., Fig 7D, since the goal is cued, the agent is trained on a subset of all possible goal locations and tested on novel goal locations. We added clarifications to both the text and figure captions :

(p.9 line 318ff) *We therefore test generalization in both guidance and aiming. In order to test generalization in guidance, the agent was trained using a subset of all possible start locations, and had to navigate to the same unmarked goal from novel start locations in the test phase. For testing generalization in aiming, the agent was trained on a subset of cued goal locations and had to navigate to novel goal locations in the test phase.*

(Caption, Fig.7)*...Generalization performance of agent in guidance (successful trials in test after being trained from different start locations)...Generalization performance of agent in aiming (successful trials in test after being trained to navigate to different cued goal locations)*

(b) If so, how were the novel goal locations chosen, and how were they distributed over the environment?

We apologize for missing this detail in the Methods. For both guidance and aiming, the novel start and goal positions were chosen randomly at the beginning of training by splitting all possible start and goal locations into training (80%) and test (20%) sets. We have now included this in the Methods, after the description of how generalization is tested :

(p.6 line 202ff.) *The training set consisted of 80% of all possible start locations for guidance, and 80% of all possible cued goal locations for aiming. The remaining 20% unseen start and goal locations were used as test sets for guidance and aiming, respectively. The assignments were made randomly for each agent before training started.*

Fig 7E shows the satisfying result that increasing regularization with dropout increases the place representations during guidance. Is this also the case for the vector-like representations during

aiming? Why is this not shown? It would be nice to see the double dissociation.

We had not included the corresponding figure for the aiming task initially because it showed a null result, i.e. a ceiling effect with a large number of vector representations emerging at all dropout levels. Following the reviewers question, we reconsidered our decision and included the new panel 7F in Fig. 7. We also added the following text:

(p.10, line 361ff.) *We however do not observe a similar effect for vector-like representations in aiming, where the proportion of these units remains stable with changing dropout. We hypothesize that this is due to a ceiling effect caused by the fact that a large number of vector-like representations are already present even at zero dropout rates, presumably because these representations are critical to solving aiming with an egocentric action space.*

Minor comments
- p5. For Q-learning, the reference should be Watkins & Dayan (1992).

We have corrected this.

- P312-314. Reference is made to fig 7C but I think this should be 7B. Also the error bars in 7B seem to overlap. Is the generalisation decay significant?

Thank you for pointing this out, we have updated the reference. The generalization decay is significant ($p<0.05$) in the medium and large environments. This is now reflected in the figure.

- What happens to the agents when they walk into a wall or corner?

The agents effectively "bounce back" in this case, that is, they remain at the same position and receive the usual step penalty of -1. We added a line to clarify this in the Methods.

(p. 4 line 98) *When the agent attempted to walk into a wall or corner, it simply remained in place and the action was penalized with a -1 negative reward.*

- The authors mention in the discussion some previous models mapping allocentric and egocentric navigation strategies to model-based and model-free RL, respectively. Note that there is some experimental evidence for that mapping. For example, Kosaki et al. (2015) showed that place learning on the plus maze is devaluation sensitive (consistent with MB RL) while response learning is not. See also Vikbladh et al. (2019).

We have added references to the above literature and slightly expanded our discussion :

(p. 14, line 556) *There is some experimental evidence supporting the mapping of these RL algorithms to the place vs. response dichotomy in rodents (Kosaki, 2018) and in humans, the hippocampus has been linked to model-based planning (Vikbladh, 2019).*

While these models approach the issue of how RL algorithms could be mapped on to a cognitive vs. behavioral navigation system, our model provides a complementary approach that looks at how different

reference frames could be used at the cognitive level, and learns to navigate with either strategy using a simple model-free algorithm.

__Reviewer #2:__

The authors presented a deep reinforcement model to study the emergence of spatial representations in allo- or ego-centric frame. Overall, the study is fine. However, the analysis is bit incomplete.

1) In Fig.4, the firing maps of the output units in the DQN are analyzed. For egocentric representations, it is likely that the distance and directions to the goal are encoded in the activities of the units. Have you tried to show the firing rate maps as a function of the distance and orientation to the goal in the egocentric frame? From fig 5, it can be seen that the percentage of other cells is quite high (~10%) in aiming task. It would be interesting to see if these cells actually encode some information.

We thank the reviewer for bringing this up. We note that vector-like units were in fact identified and classified in the analysis based on their encoding jointly the direction and distance to the goal, but we failed to show visual examples of that type of response. We have added four example to the new Fig. 4C.

We also added the activities of the "other" cell types in supplementary figure S2.

2) In simulation, the distance the agent moves within one step is not given clearly. If this distance is large, and due to the discretization, the agent may not reach the goal. The authors should clarify this point.

This is a keen observation, and was not previously explicitly discussed in the methods. To ensure that the agent could always physically navigate to the goal, the goal locations were also constrained to be placed on the nodes of the discretized environment and agents moved one link at a time. We have clarified the methods:

(p.5 line 151ff.) *Translations and heading directions were constrained such that the agent moves on a hexagonal grid (Fig. 2B). That means that the six different movement and heading directions are aligned with the main axes of the grid, each 60 degrees apart, and one movement takes the agent from current node to a neighboring node on the graph. Furthermore, the goal is always placed on a node of the grid to ensure that it can always be reached by the agent despite its motion.*

3) From Fig. 7A,C, it seems that egocentric representation is more robust than the allocentric representation. The description in the text does not match the figure, since in the text, the authors wrote:" However, the generalization performance for the egocentric strategy indeed deteriorates more than for the allocentric strategy as the arena size increases (Fig. 7C)."

We apologize for this error, the reference should have been made to Fig. 7B and not 7C, which does show the deteriorating generalization performance of the allocentric agent. The reference has been modified accordingly.

4) In order to test generalization, the authors use novel stating positions. The change introduced is somewhat small. It would be interesting to see the generalization performance with more drastic changes: train the network in small sized environment, and test the performance in large and medium-sized environments.

We thank the reviewer for this suggestion. We agree that it would have been interesting to also study generalization in other ways, however, we did not explore this within the scope of our study for two main reasons. First, the main motivation of the generalization study was to evaluate under what conditions it is advantageous to use one navigation strategy over the other, and how strategy use could potentially be experimentally determined using a simple test of generalization. Fig. 7B accomplishes that for the guidance task. Secondly, any change to the environment, even a simple one, between training and testing leads to an ambiguity in defining the “correct” location of the goal in the testing phase. For instance, if the size of the environment is scaled after training, is the new correct goal the location at the same relative distances from the walls as the goal in the previous environment, at the same absolute distance(s) to the nearest wall(s), or at the same coordinates w.r.t. some distal reference frame? While this issue is very interesting, addressing it is outside the scope of this study.
