# Supplementary material for: Navigation task and action space drive the emergence of egocentric and allocentric spatial representations
Source: PLoS Comput Biol. 2022 Oct 31;18(10):e1010320. doi: 10.1371/journal.pcbi.1010320 (PMC9648855; doi:10.1371/journal.pcbi.1010320)
Supplement: Attachment — Submitted filename: Responses to reviewers.pdf [file pcbi.1010320.s004.pdf]

We once again thank the reviewers and editors for their valuable feedback on our manuscript. Below are our responses to the editorial recommendations, according to which we have modified our manuscript.

[1] In the Introduction, "However, there three major issues with this view" should be "However, there are three major issues with this view"

Thank you for spotting the error. We have corrected it.

[2] In the Results, where you state that "...studies have shown that the use of the allocentric strategy is associated with general cognitive decline [Weniger et al., 2011]", you may wish to provide a few more details of the cited study so that the implications are clearer to the reader

We have expanded the text to add some details of the study as follows :

(p.8., line 282) Indeed, allocentric memory deficits have been observed in subjects with mild cognitive impairment (MCI). For instance, Weniger et al (2011) studied subjects' navigational abilities in a virtual reality park and maze, and found that participants with MCI were impaired in their ability to navigate in both environments as compared to controls. They also found that patients with MCI were strongly impaired in their performance on neuropsychological tests of allocentric memory. While the navigation deficits in the park seem to be associated with this impaired allocentric memory, the deficits in the maze are harder to interpret, since many participants were unable to even find a navigation strategy in this environment, again underscoring the importance of finding an appropriate strategy for a given task.

[3] When discussing whether RL may be a good model for navigation, it would seem highly relevant to mention that the length of training used here (and in other RL models of navigation) is typically much greater than that required by rodents and other mammals completing similar tasks

Thank you for the suggestion. We have addressed it in the discussion -

(p.15., line 571) It is important to note that a major difference between deep RL agents and mammals learning to navigate is the typical time required to learn a navigation task. While rodents and other mammals are typically capable of learning these tasks within a few blocks of trials, RL agents, such as ours, require hundreds to thousands of training episodes depending on task complexity.
